# Phospho-Regulation of the *Neurospora crassa* Septation Initiation Network

**DOI:** 10.1371/journal.pone.0079464

**Published:** 2013-10-21

**Authors:** Yvonne Heilig, Kerstin Schmitt, Stephan Seiler

**Affiliations:** 1 Institute for Biology II – Molecular Plant Physiology, Albert-Ludwigs University Freiburg, Freiburg, Germany; 2 Freiburg Institute for Advanced Studies (FRIAS), Albert-Ludwigs University Freiburg, Freiburg, Germany; 3 Institute for Microbiology and Genetics, University of Goettingen, Goettingen, Germany; Cancer Research UK London Research Institute, United Kingdom

## Abstract

Proper cell division is essential for growth and development of uni- and multicellular organisms. The fungal septation initiation network (SIN) functions as kinase cascade that connects cell cycle progression with the initiation of cytokinesis. Miss-regulation of the homologous Hippo pathway in animals results in excessive cell proliferation and formation of tumors, underscoring the conservation of both pathways. How SIN proteins interact and transmit signals through the cascade is only beginning to be understood. Moreover, our understanding of septum formation and its regulation in filamentous fungi, which represent the vast majority of the fungal kingdom, is highly fragmentary. We determined that a tripartite kinase cascade, consisting of CDC-7, SID-1 and DBF-2, together with their regulatory subunits CDC-14 and MOB-1, is important for septum formation in the model mold *Neurospora crassa*. DBF-2 activity and septum formation requires auto-phosphorylation at Ser499 within the activation segment and phosphorylation of Thr671 in the hydrophobic motif by SID-1. Moreover, SID-1-stimulated DBF-2 activity is further enhanced by CDC-7, supporting a stepwise activation mechanism of the tripartite SIN kinase cascade in fungi. However, in contrast to the situation described for unicellular yeasts, the localization of the entire SIN cascade to spindle pole bodies is constitutive and cell cycle independent. Moreover, all SIN proteins except CDC-7 form cortical rings prior to septum initiation and localize to constricting septa. Thus, SIN localization and activity regulation significantly differs in unicellular versus syncytial ascomycete fungi.

## Introduction

Proper cell division is essential for growth and development of uni- and multicellular organisms. The fission yeast septation initiation network (SIN) has been identified as a tripartite kinase cascade that connects cell cycle progression with the initiation of cytokinesis [1]. Miss-regulation of the homologous Hippo pathway in animals results in excessive proliferation and formation of tumors [2]. Activation of the SIN by the spindle pole body (SPB)-associated ras superfamily GTPase Spg1 results in recruitment of the STE kinase Cdc7 to SPBs. Two additional SPB-associated kinases Sid1 and Sid2, with their respective regulatory subunits Cdc14 and Mob1 are part of the SIN. Localization studies indicate a hierarchical order of Cdc7 – Sid1 – Sid2, although biochemical evidence for a linear cascade is lacking [3]. Phosphorylation of the phosphatase Clp1 by the nuclear DBF2-related (NDR) effector kinase Sid2 promotes mitotic exit by counteracting the function of the cyclin kinase Cdc2 [4]. In addition, the active Sid2-Mob1 kinase complex, yet not the other SIN components, relocates to the cell cortex, where it is involved in the assembly and constriction of the contractile actomyosin ring that triggers septum formation [5,6].

The related mitotic exit network (MEN) of budding yeast has a similar composition and functions in an analogous manner [7]. However, two significant differences exist. First, the MEN lacks a homolog of the fission yeast kinase Sid1, and the effector kinase Dbf2p is directly phosphorylated by the Cdc7 homolog Cdc15p [8]. The Dbf2p phospho-sites identified in this study correspond to sites highly conserved in other fungal nuclear NDR kinases [9,10], suggesting that Dbf2p activation involves activation segment auto-phosphorylation and phosphorylation of its C-terminal hydrophobic motif through Cdc15p. Second, budding yeast MEN mutants arrest late in the mitotic cell cycle, while the fission yeast SIN is not essential for mitotic exit, and SIN mutants generate aseptate and multinucleated cells. This is primarily based on the different morphologies of both yeasts and the fact that the MEN, but not the SIN, is an essential part of a checkpoint that monitors spindle position in anaphase [7].

Filamentous fungi represent the vast majority of the fungal kingdom. However, despite the importance of septum formation for growth and differentiation of molds, our understanding of septum formation and its regulation in molds is highly fragmentary [11,12]. In contrast to the total separation of mother and daughter cells in unicellular yeasts, the hyphal network formed by filamentous fungi is compartmentalized by incomplete septa. The septal pores enable communication and transport of metabolic components, RNA and organelles between adjacent cells. This controlled segmentation of hyphal units is the basis for the morphological complexity achieved by fungi and for the success of the fungal kingdom [13,14]. Genetic and biochemical analysis in the model molds *Neurospora crassa* and *Aspergillus nidulans* revealed that cell cycle-dependent signals of a subset of competent mitotic nuclei within the multinucleate hyphal compartments activate the SIN [15–18]. This, in turn, is critical for the cortical localization of a RHO-4 – BUD-3 GTPase module to sites of future septum formation. The RHO-4 – BUD-3 complex recruits a second RHO-4 – RGF-3 GTPase module, which is required for formin-dependent generation of the contractile actomyosin ring and its subsequent constriction [19–21].

Although most components of the SIN are present in the genomes of both model molds [22,23], a mechanistic understanding of the SIN in the multinuclear hyphal context of filamentous fungi is currently lacking. Mutants in the currently characterized *A. nidulans* SIN kinase SEPH (Cdc7 homolog) and the MOBA kinase adaptor (Mob1 homolog) are aseptate [24,25]. Similarly, the DBF-2 – MOB-1 complex is essential for septum formation in *N. crassa* [26]. The *A. nidulans* SIN proteins MOBA and SIDB (Dbf2 homolog) localize to SPBs and constricting septa [25,27]. However, in contrast to both yeasts, where SPB association of the SIN kinases is essential for activation of the NDR effector kinase and subsequent recruitment of the NDR-Mob1 complex to the cell cortex, SIN activation in *A. nidulans* does not require SPB association [27]. Already these limited data available in molds suggest major differences in the regulation of the SIN in unicellular versus syncytial fungi. Moreover, a mechanistic picture how SIN proteins interact and transmit signals through the cascade to trigger CAR assembly and constriction is only beginning to emerge in fungi. In this study, we characterized the central SIN network in *N. crassa*, and provide biochemical evidence for a stepwise activation of a tripartite kinase cascade, consisting of CDC-7, SID-1 and DBF-2.

## Results

### A tripartite SIN cascade is important for septum formation and localizes constitutively to SPBs and septa

**Figure 1:**
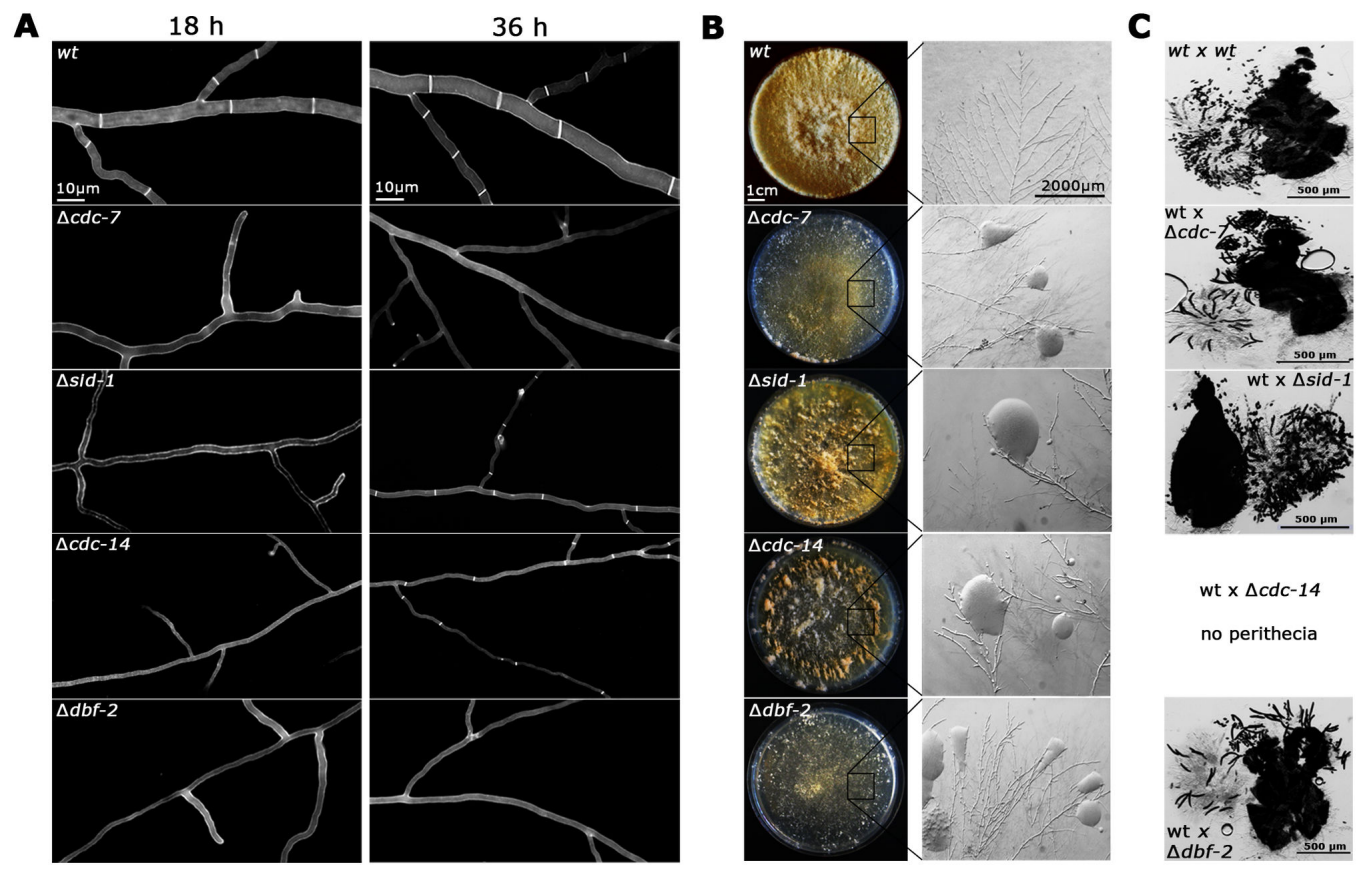
*N. crassa* SIN components are required for septum formation but display distinct mutant characteristics (**A**) Deletion strains defective in the indicated SIN components generate thin and aseptate hypha in young colonies (18 h time point). In older colonies, the septation defects were suppressed in ∆*sid-1* and ∆*cdc-14* strains (36 h time point). Cell wall and septa were labeled with Calcofluor White. (**B**) SIN mutants showed cytoplasmic leakage (magnified inserts), but, due to the fast ability to septate, ∆*sid-1* and ∆*cdc-14* generated abundant aerial mycelium and asexual spores (conidia; plate morphology). (**C**) SIN mutants displayed distinct abnormalities during sexual development. wt x ∆ crosses with ∆*cdc-7*(het) and ∆*dbf-2*(het) resulted in the frequent formation of large, banana-shaped ascospores. In contrast, wt x ∆*sid-1*(het) progeny morphology was normal, while crosses of wt x ∆*cdc-14*(het) produced no mature perithecia .

BLAST searches of the *N crassa* genome with *S. pombe* and *A. nidulans* SIN proteins identified homologs for all components of the SIN network except one of the scaffold proteins, which is poorly conserved among different species (Table 1). Mutants defective in predicted components of the tripartite kinase cascade were available as heterokaryotic strains as part of the *Neurospora* Genome project. Crosses of ∆*NCU01335* and ∆*NCU04096* with *wild type* resulted in the expected segregation of the hygromycin cassette used for the gene deletions [28], and the hygromycin-resistent progeny produced thin and aseptate hyphae, which frequently lysed (Figure 1 A, B). Thus, we concluded that NCU01335 and NCU04096 function as part of the SIN, and the proteins were designated CDC-7 and SID-1, respectively. 

**Table 1:** (Predicted) SIN components in yeasts and filamentous fungi.

Protein feature	*S. pombe*	*S. cerevisiae*	N. crassa *	A. nidulans *
Polo kinase	Plo1	Cdc5p	NCU09258	PLKA
GTPase	Spg1	Tem1p	NCU08878	AN7206
two component GAP	Cdc16	Bub2p	NCU03237	BUBA
	Byr4	Bfa1p	NCU11967	BYRA
STE kinase	Cdc7	Cdc15	NCU01335	SEPH
GC kinase	Sid1	/	NCU04096	AN8033
GC kinase adaptor	Cdc14	/	NCU06636	AN0655
NDR kinase	Sid2	DBF-2p	DBF-2	SIDB
NDR kinase adaptor	Mob1	Mob1p	MOB-1	MOBA
Leucin-rich scaffold	Cdc11	Nud1p	NCU03545	SEPK
Coiled coil scaffold	Sid4	?	?	SNAD

* Generic NCUxxxxx and ANxxxx nomenclature indicates uncharacterized proteins

As previously described for ∆*dbf-2* and ∆*mob-1* [26], ∆*cdc-7* colonies started to show sectors within 1-2 days, in which septa occurred. Also the formation of aerial hyphae and the initiation of the asexual developmental program and production of conidia were strictly dependent on the ability to form septa. Back-crosses of septum-forming ∆*cdc-7* colonies (and of ∆*sid-1* or ∆*cdc-14* colonies; see below) with *wild type* resulted in two types of hygromycin-resistent progeny: aseptate germlings that produced septa only at later stages of colony development and germlings that formed septa with frequencies that were similar to those of wild type germlings. When we compared the frequency of suppressor occurrence between the different strains, we noted that ∆*sid-1* behaved differently than ∆*cdc-7*, ∆*dbf-2* and ∆*mob-1* in that septa appeared much faster in this mutant, resulting in the formation of abundant aerial mycelium and sporulation (Figure 1 A, B). Thus, we also analyzed a deletion strain defective for its predicted regulatory subunit NCU06636/CDC-14, which is essential for Sid1 function in fission yeast [1]. ∆*cdc-14* germlings were initially aseptate, but produced septa with frequencies comparable to ∆*sid-1* and faster than the other SIN deletion strains (Figure 1 A, B). In support of different defects produced by ∆*sid-1* and ∆*cdc-14* versus ∆*cdc-7*, ∆*dbf-2* and ∆*mob-1*, we also noted that the morphology of sexual progeny generated in wt x ∆*sid*-1 crosses was normal, while wt x ∆*cdc-14* crosses did not result in mature perithecia (Figure 1 C). This contrasted with the generation of large, banana-shaped ascospores produced in wt x ∆ crosses with ∆*cdc-7* as we have previously shown for ∆*dbf-2* and ∆*mob-1* [26]. 

**Figure 2:**
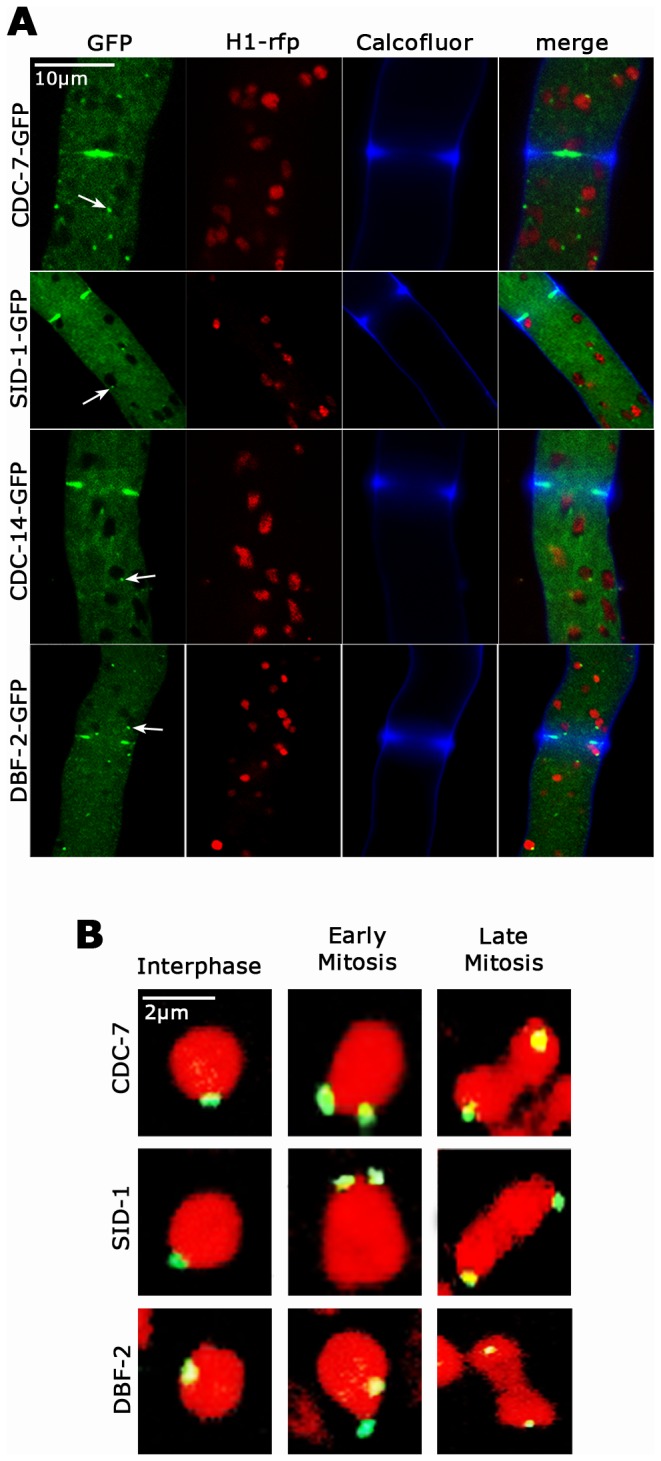
*N. crassa* SIN components localize to SPBs and septa (A) Functional GFP fusion proteins of CDC-7, SID-1, CDC-14 and DBF-2 localized to spindle pole bodies (arrows) and as constricting rings at forming septa. Nuclei were labeled with histone H1-RFP, the cell wall was stained with Calcofluor White. (**B**) The localization of the three SIN kinases CDC-7, SID-1 and DBF-2 to SPBs is constitutive and cell cycle independent. The three SIN kinases associate with SPBs of interphase nuclei as well as during early and late mitotic stages (as indicated by nuclear morphology). Nuclei were labeled with histone H1-RFP.

Next, we generated strains expressing GFP-fusion proteins to investigate the cellular distribution of these SIN proteins. All constructs were controlled by the *ccg-1* promoter and targeted to the *his-3* locus in the respective deletion strain in order to confirm functionality of the fusion proteins. In order to test for potential effects of ectopic over-expression, we also modified the endogenous locus of *dbf-2* to allow expression of DBF-2-GFP under the control of its endogenous regulatory elements. Although *ccg-1* driven GFP-DBF-2 was expressed at ca. 3-fold higher levels, resulting in enhanced cytoplasmic background fluorescence (Figure S1), we observed no differences in the localization pattern of DBF-2 in the two strains (data not shown). We determined that the entire SIN cascade associated with septa (Figure 2 A). DBF-2, SID-1 and CDC-14 accumulated first as cortical ring at the cell cortex prior to the initiation of septum constriction and remained associated with the septal pore of the mature septum after completion of the septation process (Movies S1, S2, S3). We were unable to detect CDC-7-GFP at the cell cortex and a clear CDC-7 signal was only visible at the septal pore of the mature septum. The failure to observe CDC-7 at early stages of septum formation is consistent with the low expression level of *ccg-1* driven CDC-7-GFP (Figure S1). Alternatively, this may indicate that CDC-7 only associates with the septum after constriction. Moreover, all SIN components associated with spindle pole bodies (SPBs) in a constitutive manner and independently of the cell cycle state (Figure 2 B).

### CDC-7-dependent activation of DBF-2 occurs through SID-1

**Figure 3:**
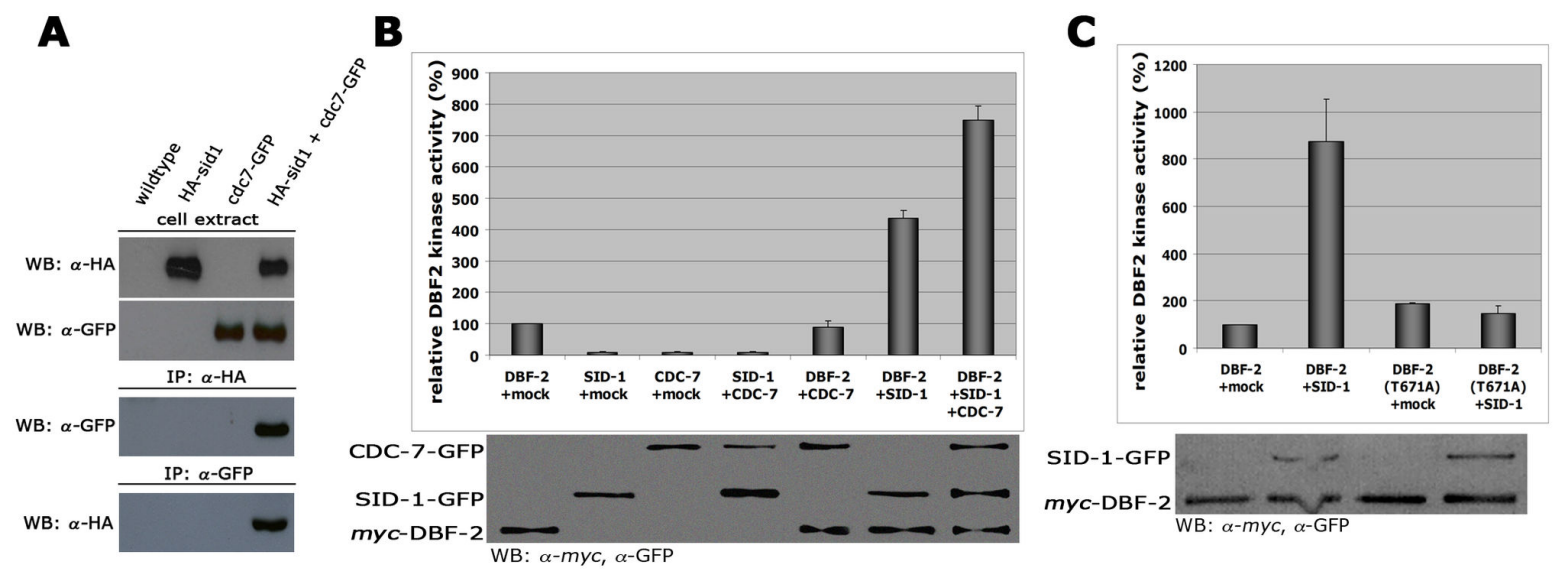
CDC-7-dependent activation of DBF-2 occurs through SID-1 (A) Reciprocal co-immunoprecipitation experiments of CDC-7-GFP and HA-SID-1 from cell extracts co-expressing both functionally tagged proteins indicate interaction of the two kinases. (**B**) *In* *vitro* DBF-2 activity assays revealed that addition of separately purified SID-1 stimulate DBF-2. SID-1-dependent stimulation of DBF-2 was further increased by addition of CDC-7 to the reaction. As control, CDC-7 alone did not stimulate DBF-2 (n = 5). To confirm equal precipitation of protein used for the kinase reactions, the kinase-sepharose pellet after the kinase reaction was boiled in Laemmli buffer and the supernatant used to determine equal protein abundance by SDS-PAGE and Western blot. (**C**) SID-1 was able to stimulate DBF-2, but not DBF-2(T671A). Western blot analysis of the precipitated proteins was used to determine comparable kinase levels (n = 5).

Activation of Dbf2p in *S. cerevisiae* involves direct phosphorylation by the Cdc7 homolog Cdc15p [8]. In contrast, the *S. pombe* SIN cascade likely acts in the order Cdc7-Sid1-Sid2, but biochemical evidence for a step-wise activation of these kinases is lacking [3,29]. In order to determine the functional relationship between the three *N. crassa* SIN kinases and to provide a mechanism for DBF-2 activation, we tested if CDC-7 interacted with SID-1 and/or DBF-2. Reciprocal co-immunoprecipitation (co-IP) experiments of CDC-7-GFP and HA-SID-1 from cell extracts co-expressing both functionally tagged proteins proved a stable interaction of the two kinases (Figure 3 A). In contrast, we were unable to show interactions between CDC-7 and DBF-2 or SID-1 and DBF-2 in co-IP experiments performed under identical conditions (data not shown), suggesting that these interactions may be more dynamic. 

Wild type DBF-2 precipitated from *N. crassa* extracts displayed activity towards a synthetic peptide encompassing the consensus NDR kinase target motif, which we had previously used for *in vitro* activity assays with the related NDR kinase COT-1 [30]. Using this assay, we determined that precipitated SID-1 enhanced the activity of separately purified DBF-2 (Figure 3 B). If the *N. crassa* SIN functions as tripartite, stepwise kinase cascade, we hypothesized that SID-1-dependent stimulation of DBF-2 should be further increased by addition of CDC-7 – a prediction that we were able to confirm in *in vitro* assays of individually precipitated components (Figure 3 B). Control experiments with SID-1 or CDC-7 precipitates proved the specificity of this assay for the NDR kinase and support the interpretation that that SID-1 is required for transmitting CDC-7-dependent signals towards DBF-2.

Activation of NDR kinases requires phosphorylation of a C-terminal hydrophobic motif [9,31,32]. We determined that SID-1-dependent stimulation of DBF-2 was only possible with purified wild type DBF-2, but not with DBF-2(T761A), which contained a threonine to alanine substitution of the predicted hydrophobic motif phosphorylation site (Figure 3 C). Phosphorylation experiments coupled with mass-spectrometric analysis further supported Thr671 phosphorylation of DBF-2 by SID-1: we were only able to identify phosphopeptides of the hydrophobic motif of DBF-2 precipitated under high-stringency conditions when we co-incubated DBF-2 with separately purified SID-1 in *in vitro* kinase reactions (Figure S2). Based on these results, we conclude that SID-1 phosphorylates DBF-2 at Thr671. 

### Dual phosphorylation of DBF-2 is required for kinase activity and septum formation

**Figure 4:**
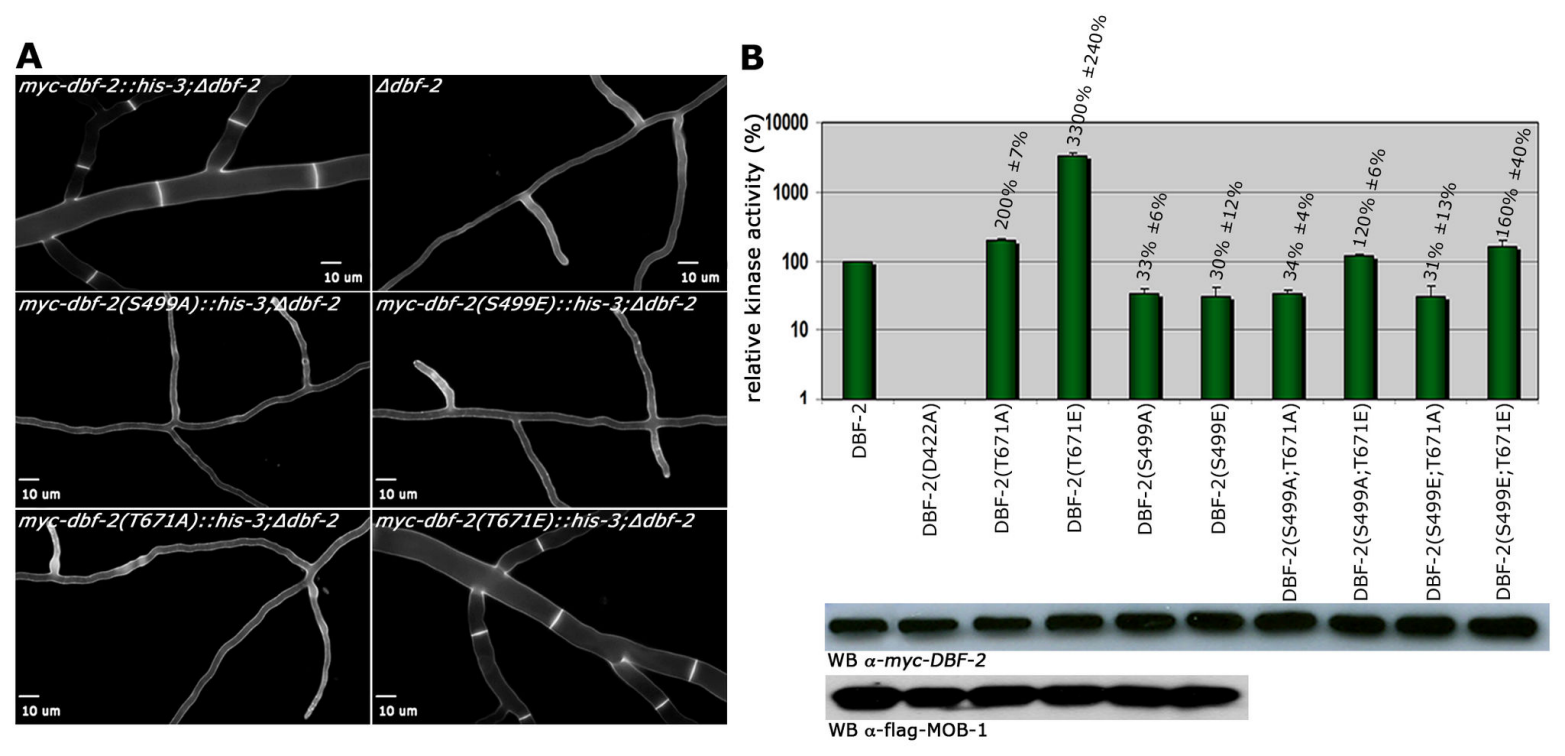
Dual phosphorylation of DBF-2 is required for kinase activity and septum formation. (**A**) Functional characterization of two conserved phosphorylation sites of DBF-2. The phosphomimetic DBF-2(T671E) variant complemented Δ*dbf-2*, while substitution of Ser499 to alanine and glutamate and Thr671 to alanine did not. Cell wall and septa were labeled with Calcofluor White. (**B**) Kinase activity and MOB-1 interaction pattern of the indicated DBF-2 variants. Hydrophobic motif phosphorylation of Thr671 was required for maximal kinase activity, while either modification of Ser499 within the activation segment reduced DBF-2 activity to ca. 30% of the wild type DBF-2 control. Phospho-site double mutant analysis indicated that substitution of Thr671 to glutamate in a S499A and S499E background could only partly restore kinase activity. Precipitated DBF-2 variants were assayed *in* *vitro* using the synthetic NDR kinase peptide (KKRNRRLSVA) as substrate (n = 5). Western Blot analysis indicated equal precipitation of the co-activator protein MOB-1 with DBF-2 activation segment and hydrophobic motif variants.

In order to further dissect the phospho-regulation of DBF-2, we characterized strains expressing DBF-2 variants harboring point mutations in the predicted auto-phosphorylation and hydrophobic motif sites Ser499 and Thr671, respectively. Both, the Ser499 to alanine (inactive mimic) and to glutamate (active mimic) substitutions were nonfunctional, and these kinase variants were unable to complement the septation defects of ∆*dbf-2* (Figure 4 A). Of the Thr671 to alanine/glutamate substitutions, DBF-2(T671E), but not DBF-2(T671A) was functional and complemented the deletion strain. DBF-2 variants harboring either of the two Ser499 substitutions displayed *in vitro* kinase activities reduced to ca. 1/3 of the wild type DBF-2 control (33±6% and 30±12% for DBF-2(S499A) and DBF-2(S499E), respectively; n = 5; Figure 4 B). In contrast, the kinase activity of DBF-2(T671A) was slightly increased (200±7%; n = 5), while DBF-2(T671E) displayed >30-fold increased activity (3300±240%; n = 5). Modification of homologous residues affected the interaction of the *S. pombe* NDR kinase Sid2 with Mob1 [29]. However, MOB-1 binding was not affected by these modifications in *N. crassa*, and wild type MOB-1 levels co-precipitated with each DBF-2 variant (Figure 4 B). We also performed *in vitro* phosphorylation experiments coupled with mass-spectrometric analysis of kinase variants precipitated under high-stringency conditions to confirm Ser499 as auto-phosphorylation site. Strong phosphophorylation of Ser499 in tryptic peptides generated from wild type DBF-2 and hyperactive DBF-2(T671E), but not kinase-dead DBF-2(D422A) indicated that Ser499 is the primary site of DBF-2 auto-phosphorylation (Figure S2). Moreover, we noticed phosphorylation of multiple S/T residues in the N-terminal, non-catalytic region of the kinase in DBF-2(T671E).

The fact that both Ser499 modifications reduced DBF-2 activity *in vitro* and were nonfunctional *in vivo* may suggest that dynamic modification of the activation segment (i.e. the regulated phosphorylation/dephosporylation) could modulate DBF-2 function. We generated various combinations of double mutants in order to further explore this possibility (Figure 4 B). None of these double modifications resulted in functional protein, and complementation of the ∆*dbf-2* defects failed with all constructs (data not shown). Kinase assays revealed that the two Thr671 to alanine variants DBF-2(S499A;T671A) and DBF-2(S499E;T671A) displayed reduced activities, which were similar to the individual Ser499 mutations (34±4% and 31±13% of wild type DBF-2, respectively; n = 5). Substitution of the hydrophobic motif threonine with glutamate in a S499A and S499E background increased kinase activity 3.6- and 5.3-fold compared to the respective Ser499-modified protein (n = 5). Thus, phosphorylation of Thr671 can partly overcome the lack of activation segment modification, but the stable modification of Ser499 prevented full activation of DBF-2.

## Discussion

Regulation of septum formation and the composition of the SIN in filamentous fungi is only beginning to be unraveled. The phenotypic and biochemical analysis of predicted SIN components allowed the identification of the SIN kinase cascade CDC-7, SID-1 and DBF-2 together with their respective regulatory subunits CDC-14 and MOB-1, and the characterization of their hierarchical relationship in *N. crassa*. Based on *in vitro* kinase assays, we provide the first biochemical evidence that SID-1 activates DBF-2 through hydrophobic motif phosphorylation, analogous to POD-6-dependent phosphorylation of COT-1 [32,33] or activation of related fungal and animal NDR kinases through upstream germinal centre kinases of the STE kinase family [9,31,34,35]. We observed no direct activation of DBF-2 by CDC-7, but SID-1-dependent stimulation of DBF-2 was further enhanced through CDC-7. Although our data do not rule out that direct phosphorylation of DBF-2 by CDC-7 is a prerequisite for hydrophobic motif phosphorylation of DBF-2 by SID-1, we consider this interpretation unlikely. It was previously shown that the budding yeast kinase Cdc15p also targets the homologous threonine residue present in the hydrophobic motif of Dbf2p [9,31,34,35], just as we have determined for SID-1-dependent phosphorylation of DBF-2 in *N. crassa*. Thus our data, strongly suggest that the *N. crassa* SIN functions as hierarchical, stepwise kinase cascade.

Interestingly, we observed that strains carrying deletions of *sid-1* or its regulatory subunit *cdc-14* behaved differently than mutants in the remaining SIN components. Although initially aseptate, accumulation of suppressor mutations and, as result, the capability to form septa and to produce mitotic conidiospores was much higher in ∆*sid-1* and ∆*cdc-14* than in other *SIN* mutants (although all *SIN* mutants regain the ability to form septa at some point). Moreover, ∆*sid-1* and ∆*cdc-14* progeny did not form the large, banana-shaped ascospores described for ∆*dbf-2*, ∆*cdc-7* and ∆*mob-1* containing crosses (this study [26]), suggesting that the presentation of the SIN as a linear kinase cascade may represent a simplified view. Possible scenarios could be that, in contrast to our *in vitro* data, CDC-7 is able to directly target DBF-2 *in vivo*. Alternatively, additional uncharacterized kinases may function in concert with SID-1 to regulate DBF-2.

Our characterization of DBF-2 variants in predicted regulatory phosphorylation sites indicates that DBF-2 is regulated by dual phosphorylation. We determined that Ser499 within the activation segment is auto-phosphorylated, while the hydrophobic motif site Thr691 is targeted by the upstream kinase SID-1. Interestingly, we also detected tyrosine-specific phospho-peptides in our mass-spectrometry analysis of hyperactive DBF-2(T671E), suggesting that DBF-2 (and possibly other NDR kinases) can function as dual-specific kinases. These data are consistent with a recent report on tyrosine kinase activity of the plant NDR kinase PLK01 [36]. 

Current models for NDR kinase activity regulation predict the formation of inactive, competent and active conformations, which correspond to non-phosphorylated, auto-phosphorylated and dual-phosphorylated states, respectively [10,37]. In this study, we analyzed, for the first time, the full complement of kinase variants harboring individual and double mutant substitutions in these two regulatory sites. While DBF-2(S499A) displayed *in vitro* activity reduced to ca. 1/3^rd^ of the wild type control, DBF-2(T671A) produced activities in the range of wild type DBF-2. Thus, auto-but not hydrophobic motif phosphorylation is required for normal kinase activity. However, alanine substitution of both sites resulted in nonfunctional protein, indicating that phosphorylation of both sites is essential for the *in vivo* functionality of DBF-2. This conclusion is supported by DBF-2(T671E), which resulted in maximal *in vitro* activity and was the only DBF-2 variant functional *in vivo*. 

We further determined that glutamate substitution of the hydrophobic motif site in glutamate- and alanine-substituted auto-phosphorylation backgrounds only partially recovered *in vitro* activities and that these kinase variants were nonfunctional *in vivo*. The fact that phosphorylation of Thr671 can only partly overcome the lack of activation segment modification may suggest that dynamic modification of the activation segment may be important for full activation and functionality of DBF-2. Alternatively, Ser499 modification may simply impair the functionality of the protein. However, we consider this unlikely, because analogous substitutions have successfully been used for the analysis of several fungal [8,9,30,32,35] as well as animal [31,34] NDR kinases.

Functional GFP fusion constructs of the three SIN kinases localize to SPBs and septa. In contrast to the situation in both unicellular yeast models [3,7], SPB association of the SIN cascade is not cell cycle dependent. Moreover, SID-1 and DBF-2 localize to the cell cortex prior to septum constriction and to the forming septum. We detected CDC-7 only at the septal pore of the mature septum, possibly indicating that this kinase only associates with the septum after constriction. Alternatively, CDC-7 levels below our imaging resolution may associate with the other SIN kinases during early stages of septation. Together with the finding that SIN activation in *A. nidulans* does not require SPB association of the NDR kinase SIDB [27], these data indicate major differences in the regulation of the SIN in unicellular versus syncytial ascomycetes.

In contrast to a previous report [38], we did not detect defects of *SIN* mutants in the proper completion of the cell cycle. This observation is in line with the fact that cell cycle progression was unaffected in *A. nidulans SIN* mutants [20,24,25]. Moreover, meiotic cell divisions are not affected in *N. crassa SIN* mutants. Re-sequencing of an old laboratory strain identified a mutant called *Banana* as *dbf-2* deletion strain [39]. Its previous characterization had revealed that the eight nuclei derived from the two meiotic and one mitotic divisions are formed in a normal manner in *Ban+/Ban* asci, but that the resulting nuclei are then enclosed in a single giant ascospore [40,41]. This is consistent with data obtained for budding and fission yeasts, where the SIN is largely dispensable during meiosis, but required for spore wall formation and ascospore morphology [42,43]. In summary, these data indicate an essential function of the SIN during cross wall formation in vegetative cells and during the formation of asco- as well as conidiospores in unicellular and filamentous ascomycetes. 

## Materials and Methods

### Strains, media and growth conditions

Strains used in this study are listed in Table S1. General genetic procedures and media used in the handling of *N. crassa* are available through the Fungal Genetic Stock Center (www.fgsc.net; [44]). 

### Plasmid construction and fungal expression of tagged proteins

To obtain strains that express GFP/myc/HA-tagged fusion proteins from the *his-3* locus, the open reading frames of DBF-2, SID-1, CDC-14 and CDC-7 were amplified by PCR as annotated by the *N. crassa* database using primers listed in Table S2 and introduced via *SgsI/PacI*, *BcuI/PacI* or *XbaI/PacI*, respectively, into pCCG1_N-GFP, pMF272, pHAN1 or pCCG1-3xMYC [41,45–47]. Point mutated versions of myc-DBF-2 were generated by site-directed mutagenesis according to the manufacturer’s instructions (Stratagene). Resulting plasmids were transformed into *his-3*, *nic-3;his-3* or *trp-1;his-3*, and were selected for complementation of the *his-3* auxotrophy. Strains expressing the fusion constructs were crossed with the respective deletion strain. Progeny were selected on minimal media containing hygromycin and assayed for complementation of the mutant’s growth/septation defects. 

### Microscopy

Low magnification documentation of fungal hyphae or colonies was performed as described [48,49] using an SZX16 stereomicroscope, equipped with a Colorview III camera and Cell^D^ imaging software (Olympus). Images were further processed using Photoshop CS2 (Adobe). An inverted Axiovert Observer Z1 microscope (Zeiss) equipped with a CSU-X1 A1 confocal scanner unit and a QuantEM 512SC camera (Photometrics) was used for spinning disk confocal microscopy [50,51]. Slidebook 5.0 software (Intelligent Imaging Innovations) was used for image/movie acquisition, deconvolution and image analysis. Cells wall and plasma membrane were stained with Calcofluor white (2µg/ml) and FM4-64 (1µg/ml) respectively. Time-lapse imaging was performed at capture intervals of 20-30 s for periods up to 15 min using the oil immersion objective 100x/1.3. Image series were converted into movies (*.movs). Aseptate deletion strains were maintained as heterokaryons to avoid the accumulation of suppressor mutations. Image analysis was performed on germlings and young colonies developing from out-crossed hygromycin-resistent ascospores displaying all characteristics of the deletion stain.

### Protein methods

Liquid *N. crassa* cultures were grown at room temperature, harvested gently by filtration using a Büchner funnel and ground in liquid nitrogen. The pulverized mycelium was mixed 1:1 with IP-Buffer (50 mM Tris/HCL pH 7.5, 100 mM KCl, 10 mM MgCl_2_, 0.1% NP-40, 5 mM NaF, 1 mM PEFAbloc SC, 2mM DTT, 1 mM Na_3_VO_4_, 25 mM β-glycerophosphate, 2 mM benzamidine, 2 ng/µl pepstatin A, 10 ng/µl aprotinin, 10 ng/µl leupeptin) and centrifuged as described [32,52]. Co-immunoprecipitation was performed as described [26] with cell extracts from fused, heterokaryotic strains that were selected by their ability to grow on minimal media lacking supplements. Protein-decorated beads were washed twice with “low-stringency” IP buffer (50 mM Tris/HCL pH 7.5, 100 mM KCl, 10 mM MgCl_2_, 0.1% NP-40, 5 mM NaF, 1 mM PEFAbloc SC, 2mM DTT, 1 mM Na_3_VO_4_, 25 mM β-glycerophosphate, 2 mM benzamidine, 2 ng/µl pepstatin A, 10 ng/µl aprotinin, 10 ng/µl leupeptin), and immunoprecipitated proteins were recovered by boiling the beads for 10 min at 98°C in 3x Laemmli buffer and separated by 10% SDS-PAGE. Monoclonal mouse α-HA (clone HA-7, Sigma Aldrich), α-FLAG M2 (Sigma-Aldrich), α-myc (9E10, Santa Cruz) and α-GFP (B2, Santa Cruz) and GFP trap beads (Chromotek) were used in this study. 

DBF-2 kinase activity assays were performed as described [30,32] according to a modified protocol described previously for animal NDR kinase [53]. Total protein levels of cell extracts were determined by Bradford analysis with bovine serum albumin standard solutions as a reference, using Roti®-Quant (Carl Roth) and a Tecan Infinite® M200 microplate reader (Tecan) and adjusted with IP buffer. Recovered kinase-bound beads were washed twice with “high-stringency” IP buffer containing 0,5 M NaCl followed by two washes with kinase reaction buffer (20 mM Tris pH 7.5, 10 mM MgCl_2_, 1 mM DTT, 1 mM benzamidine, 1 mM Na_3_VO_4_, 5 mM NaF) to remove all potentially interacting (and thus co-purifying) proteins, and were finally resuspended in 50 μl kinase buffer containing 2 mM of synthetic substrate peptide (KKRNRRLSVA), 0.5 mM ATP and 1 μCi [^32^P]ATP. Western blot experiments performed as controls confirmed that no other kinase of the SIN except the tagged and precipitated protein co-purified under these “high-stringency” wash conditions. After incubation for 1 h at 37°C, samples were centrifuged for 2 min at 4.000 g, the supernatant was spotted onto P81 phosphocellulose paper circles (Whatman). Dried circles were washed 2 times with 1% phosphoric acid before incorporation of phosphate into the substrate peptide was measured by liquid scintillation counting. The remaining protein-sepharose pellet was boiled for 10 min in 3x Laemmli buffer and the supernatant was used to determine equal DBF-2 concentration in the kinase reaction by SDS-PAGE and Western blot. DBF-2 stimulation was assayed with separately purified SID-1 and CDC-7 (washed with “high-stringency” IP buffer to eliminate co-purifying contaminations), which were added during the first washing step, before kinase reaction. 

### Mass spectrometry, database analysis and enrichment of phosphopeptides

For protein identification by mass spectrometry, peptides of in-gel trypsinated proteins were extracted from of Commassie-stained gel slices as described [54]. Peptides of 5 μl sample solution were trapped and washed with 0.05 % trifluoroacetic acid on an Acclaim^®^ PepMap 100 column (75 μm x 2 cm, C18, 3 μm, 100 Å, P/N164535 Thermo Scientific) at a flow rate of 4 μl/min for 12 min. Analytical peptide separation by reverse phase chromatography was performed on an Acclaim^®^ PepMap RSLC column (75 μm x 15 cm, C18, 3 μm, 100 Å, P/N164534 Thermo Scientific) running a gradient from 96 % solvent A (0.1 % formic acid) and 4 % solvent B (acetonitrile, 0.1 % formic acid) to 50 % solvent B within 25 min at a flow rate of 250 nl/min (solvents and chemicals: Fisher Chemicals). Peptides eluting from the chromatographic column were on-line ionized by nano-electrospray using the Nanospray Flex Ion Source (Thermo Scientific) and transferred into the mass spectrometer. Full scans within m/z of 300-1850 were recorded by the Orbitrap-FT analyzer at a resolution of 60.000 at m/z 400. Each sample was analyzed using two different fragmentation techniques applying a data-dependent top 5 experiment: collision-induced decay with multistage activation and readout in the LTQ Velos Pro linear ion trap, and higher energy collision dissociation and subsequent readout in the Orbitrap-FT analyzer. LC/MS method programming and data acquisition was performed with the software XCalibur 2.2 (Thermo Fisher). Orbitrap raw files were analyzed with the Proteome Discoverer 1.4 software (Thermo Scientific) using the Mascot and Sequest search engines against the N. crasssa protein database with the following criteria: peptide mass tolerance 10 ppm, MS/MS ion mass tolerance 0.8 Da, and up to two missed cleavages allowed. Enrichment of phosphopeptides was performed based on the method developed by [55], using TiO_2_ columns (TopTip TiO_2_ 10-200 μl; Glygen Corporation). 

## Supporting Information

Figure S1 **Expression analysis of the used GFP fusion constructs under the control of the ccg-1 and their native promoters.** Anti-GFP Western blot of normalized cell extracts of strains expressing GFP fusion proteins under the control of the indicated promoters (left panel). Quantification of the relative expression levels of the indicated proteins. Protein levels are normalized to DBF-2 abundance (n = 3).
(TIF)

Figure S2 **Mass-spectrometric analysis of DBF2 phosphorylation sites.** (**A**) *In* *vitro* phosphorylation experiments coupled with mass-spectrometric analysis. Ser499 was phosphorylated in wild type DBF-2 and hyperactive DBF-2(T671E), but not DBF-2(D422A), identifying this residue as primary site of auto-phosphorylation. Multiple additional S/T phosphorylation sites were detected in the N-terminal region of DBF-2(T671E). Mass-spectrometric analysis of SID-1-dependant DBF-2 phosphorylation sites identified T671 as primary site of phosphorylation and additional S/T phosphorylation sites variously distributed. (**B**) Tryptic peptides generated from wild type DBF-2 displayed HCD-fragmentation spectrum of the peptide SIVGSPDYMAPEVLR with Ser499 phosphorylated. Fragment b-ions (yellow) and y-ions (blue) with an asterisk indicate neutral loss of ammonia (-17 Da), and ions labeled with a circle neutral loss of water water (-18 Da), charge states are in brackets. The peptide cross-correlation score for Sequest (XCorr) was 4.9 and the Mascot IonScore was 77. The probability of Ser499-phosphorylation was caclulated by the pRS algorithm to be 99.99% [J Proteome Res 10: 5354-5362]. (**C**) Representative fragmentation spectrum of the peptide SLFVGFtFR with phosphorylation at T671. Fragment ions b* and y* are ions with loss of ammonia (-17 Da), and fragment ions bo and co are ions with loss of water (-18 Da). The number in brackets indicates the charge state of the fragment ion. The peptide was fragmented by CID and fragment ions were detected in the linear ion trap. The peptide was identified with Mascot and Sequest search engines (peptide IonScore of 30 and XCorr of 3.45, respectively). pRS score for phosporylation at tyrosine was 100% [J Proteome Res 10: 5354-5362].
(TIF)

Table S1 ***N. crassa* strains used in this study.**
(DOCX)

Table S2 **Primers used in this study.**
(DOCX)

Movie S1 **Time-course of DBF-2-GFP localization during septum formation.** DBF-2-GFP formed cortical rings at incipient septation sites that constricted during septum formation and accumulated around the septal pore of the completed septum (**a**) GFP channel; (**b**) RFP channel; (**c**) merged. The plasma membrane was stained with FM4-64. Images were captured at 20 sec intervals.
(MOV)

Movie S2 **Time-course of SID-1-GFP localization during septum formation.** SID-1-GFP formed cortical rings at incipient septation sites that constricted during septum formation and accumulated around the septal pore of the completed septum (**a**) GFP channel; (**b**) RFP channel; (**c**) merged. The plasma membrane was stained with FM4-64. Images were captured at 20 sec intervals.
(MOV)

Movie S3 **Time-course of CDC-14-GFP localization during septum formation.** CDC-14-GFP formed cortical rings at incipient septation sites that constricted during septum formation and accumulated around the septal pore of the completed septum (**a**) GFP channel; (**b**) RFP channel; (**c**) merged. The plasma membrane was stained with FM4-64. Images were captured at 20 sec intervals.
(MOV)
