# Supplementary figures and images for: Phospho-Regulation of the Neurospora crassa Septation Initiation Network
Source: PLoS One. 2013 Oct 21;8(10):e79464. doi: 10.1371/journal.pone.0079464 (PMC3804505; doi:10.1371/journal.pone.0079464)

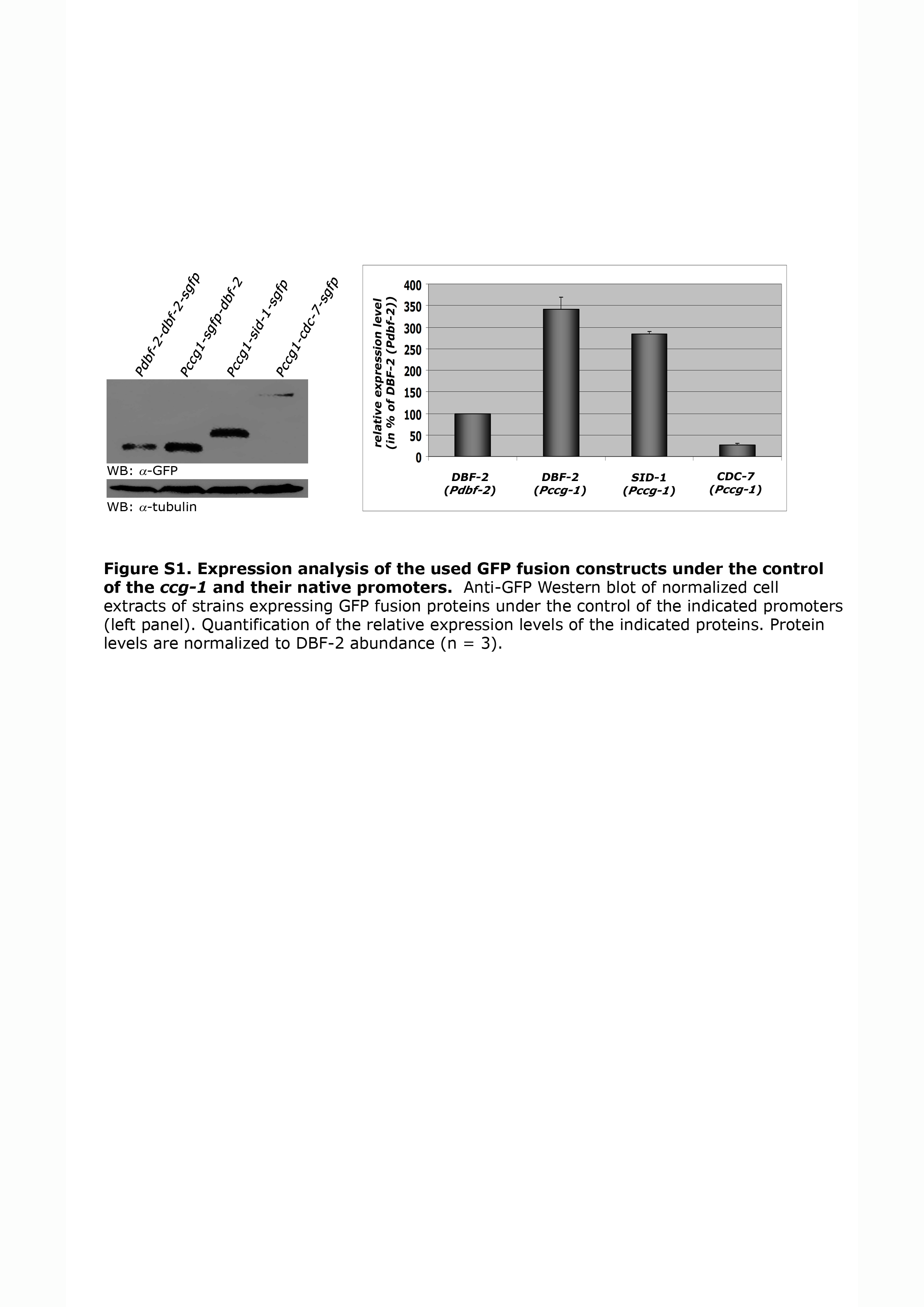

Supplement: Figure S1 — Expression analysis of the used GFP fusion constructs under the control of the ccg-1 and their native promoters. Anti-GFP Western blot of normalized cell extracts of strains expressing GFP fusion proteins under the control of the indicated promoters (left panel). Quantification of the relative expression levels of the indicated proteins. Protein levels are normalized to DBF-2 abundance (n = 3). (TIF) [file pone.0079464.s001.tif]

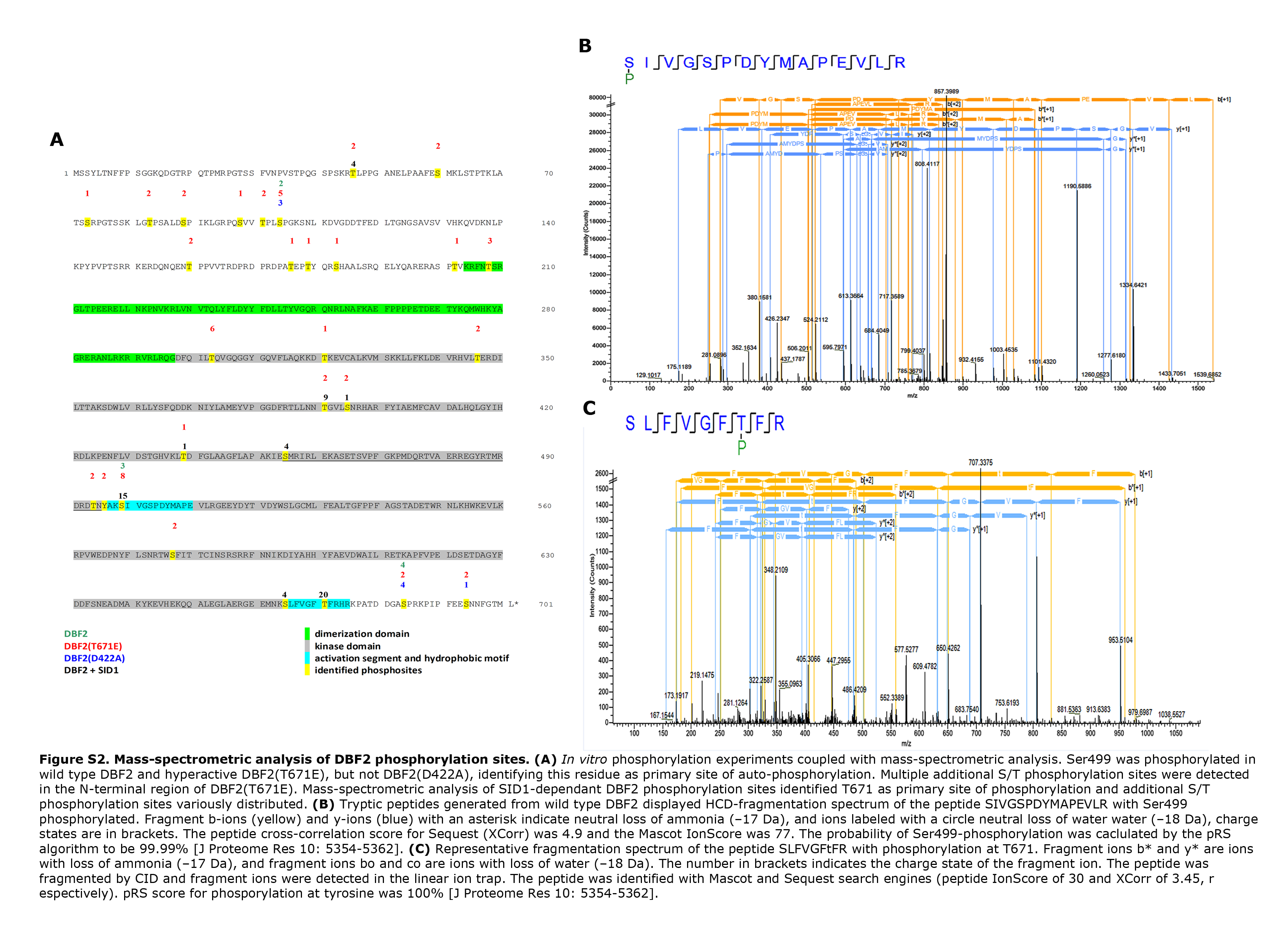

Supplement: Figure S2 — Mass-spectrometric analysis of DBF2 phosphorylation sites. (A) In vitro phosphorylation experiments coupled with mass-spectrometric analysis. Ser499 was phosphorylated in wild type DBF-2 and hyperactive DBF-2(T671E), but not DBF-2(D422A), identifying this residue as primary site of auto-phosphorylation. Multiple additional S/T phosphorylation sites were detected in the N-terminal region of DBF-2(T671E). Mass-spectrometric analysis of SID-1-dependant DBF-2 phosphorylation sites identified T671 as primary site of phosphorylation and additional S/T phosphorylation sites variously distributed. (B) Tryptic peptides generated from wild type DBF-2 displayed HCD-fragmentation spectrum of the peptide SIVGSPDYMAPEVLR with Ser499 phosphorylated. Fragment b-ions (yellow) and y-ions (blue) with an asterisk indicate neutral loss of ammonia (-17 Da), and ions labeled with a circle neutral loss of water water (-18 Da), charge states are in brackets. The peptide cross-correlation score for Sequest (XCorr) was 4.9 and the Mascot IonScore was 77. The probability of Ser499-phosphorylation was caclulated by the pRS algorithm to be 99.99% [J Proteome Res 10: 5354-5362]. (C) Representative fragmentation spectrum of the peptide SLFVGFtFR with phosphorylation at T671. Fragment ions b* and y* are ions with loss of ammonia (-17 Da), and fragment ions bo and co are ions with loss of water (-18 Da). The number in brackets indicates the charge state of the fragment ion. The peptide was fragmented by CID and fragment ions were detected in the linear ion trap. The peptide was identified with Mascot and Sequest search engines (peptide IonScore of 30 and XCorr of 3.45, respectively). pRS score for phosporylation at tyrosine was 100% [J Proteome Res 10: 5354-5362]. (TIF) [file pone.0079464.s002.tif]
