# Supplementary material for: Phospho-Regulation of the Neurospora crassa Septation Initiation Network
Source: PLoS One. 2013 Oct 21;8(10):e79464. doi: 10.1371/journal.pone.0079464 (PMC3804505; doi:10.1371/journal.pone.0079464)
Supplement: Table S1 — N. crassa strains used in this study. (DOCX) [file pone.0079464.s003.docx]

**Table S1: *N. crassa* strains used in this study**

| *Strains* | *Genotype* | Source |
| --- | --- | --- |
| *wild type 74* | *OR231 Mat A* | FGSC #987 |
| *wild type ORS* | *SL6 Mat a* | FGSC #4200 |
| *his-3 A* | *his-3 Mat A* | FGSC #6103 |
| *his-3 a* | *his-3 Mat a* | FGSC #718 |
| *trp-1;his-3* | *trp-1- his-3-* | März et al., 2009 |
| *nic-3;his-3* | *nic-3- his-3-* | März et al., 2009 |
| *∆dbf-2* | *hph::dbf-2∆ his-3-* | März et al., 2009 |
| *∆cdc-7* | *hph::cdc-7∆ + cdc-7^+^ bar::mus-51∆* | FGSC #16741 |
| *∆cdc-14* | *hph::cdc-14∆* | FGSC #12648 |
| *∆sid-1* | *hph::sid-1∆ + sid-1^+^ bar::mus-51∆* | K. Borkovich, USA |
| *gfp-dbf-2* | *Pccg-1-sgfp-dbf-2::his-3 hph::dbf-2∆* | This study |
| *gfp-dbf-2(D422A)* | *Pccg-1-sgfp-dbf-2(D422A)::his-3* | This study |
| *gfp-dbf-2(T671E)* | *Pccg-1-sgfp-dbf-2(T671E)::his-3 hph::dbf-2∆* | This study |
| *sid-1-gfp* | *Pccg-1-sid-1-sgfp::his-3 hph::sid-1∆* | This study |
| *cdc-14-gfp* | *Pccg-1-cdc-14-sgfp::his-3 hph::cdc-14∆* | This study |
| *cdc-7-gfp* | *Pccg-1-cdc-7-sgfp::his-3 hph::cdc-7∆* | This study |
| *h1-rfp* | *Pccg-1-rfp-h1::his-3 mat* A | M. Freitag, USA |
| *myc-dbf-2* | *Pccg-1-myc-dbf-2::his-3 hph::dbf-2∆* | This study |
| *myc-dbf-2(D422A)* | *Pccg-1-myc-dbf-2(D422A)::his-3 hph::dbf-2∆* | This study |
| *myc-dbf-2(S499A)* | *Pccg-1-myc-dbf-2(S499A)::his-3 hph::dbf-2∆* | This study |
| *myc-dbf-2(S499E)* | *Pccg-1-myc-dbf-2(S499E)::his-3 hph::dbf-2∆* | This study |
| *myc-dbf-2(T671A)* | *Pccg-1-myc-dbf-2(T671A)::his-3 hph::dbf-2∆* | This study |
| *myc-dbf-2(T671E)* | *Pccg-1-myc-dbf-2(T671E)::his-3 hph::dbf-2∆* | This study |
| *myc-dbf-2(S499A/T671A)* | *Pccg-1-myc-dbf-2(S499A/T671A)::his-3 hph::dbf-2∆* | This study |
| *myc-dbf-2(S499A/T671E)* | *Pccg-1-myc-dbf-2(S499A/T671E)::his-3 hph::dbf-2∆* | This study |
| *myc-dbf-2(S499E/T671A)* | *Pccg-1-myc-dbf-2(S499E/T671A)::his-3 hph::dbf-2∆* | This study |
| *myc-dbf-2(S499E/T671E)* | *Pccg-1-myc-dbf-2(S499E/T671E)::his-3 hph::dbf-2∆* | This study |
| *HA-sid-1* | *Pccg-1-HA-sid-1::his-3 hph::sid-1∆* | This study |
| *myc-dbf-2 trp-1* | *Pccg-1-myc-dbf-2::his-3 trp-1* | This study |
| *myc-dbf-2(D422A) trp-1* | *Pccg-1-myc-dbf-2(D422A)::his-3 trp-1* | This study |
| *myc-dbf-2(S499A) trp-1* | *Pccg-1-myc-dbf-2(S499A)::his-3 trp-1* | This study |
| *myc-dbf-2(S499E) trp-1* | *Pccg-1-myc-dbf-2(S499E)::his-3 trp-1* | This study |
| *myc-dbf-2(T671A) trp-1* | *Pccg-1-myc-dbf-2(T671A)::his-3 trp-1* | This study |
| *myc-dbf-2(T671E) trp-1* | *Pccg-1-myc-dbf-2(T671E)::his-3 trp-1* | This study |
| *FLAG-mob-1 nic-1* | *Pccg-1-FLAG-mob-1::his-3 nic-1* | März et al., 2009 |
| *HA-sid-1;trp-1* | *Pccg-1-HA-sid-1::his-3 trp-1-* | This study |
| *cdc-7-gfp;nic-1* | *Pccg-1-cdc-7-gfp::his-3 nic-1-* | This study |
