# Supplementary material for: Phospho-Regulation of the Neurospora crassa Septation Initiation Network
Source: PLoS One. 2013 Oct 21;8(10):e79464. doi: 10.1371/journal.pone.0079464 (PMC3804505; doi:10.1371/journal.pone.0079464)
Supplement: Table S2 — Primers used in this study. (DOCX) [file pone.0079464.s004.docx]

**Table S2. Primers used in this study**

| **Name** | **Sequence 5‘- 3‘** | |  |
| --- | --- | --- | --- |
| point mutation constructs: | |  | |
| YH-DBF2-D2A-forw | CTG GGT TAC ATT CAT CGC *gca* CTC AAG CCG GAG AAC TTC C | |  |
| YH-DBF2-D2A-rev | GGA AGT TCT CCG GCT TGA G*tg* *c*GC GAT GAA TGT AAC CCA G | |  |
| YH-DBF2-T2E-forw | GAG CTT GTT TGT CGG ATT C*ga g*TT CCG TCA TCG CAA GCC GG | |  |
| YH-DBF2-T2E-rev | CCG GCT TGC GAT GAC GGA A*ct c*GA ATC CGA CAA ACA AGC TC | |  |
| YH-DBF2-T2A-forw | GAG CTT GTT TGT CGG ATT C*gc c*TT CCG TCA TCG CAA GCC GG | |  |
| YH-DBF2-T2A-rev | CCG GCT TGC GAT GAC GGA Agg cGA ATC CGA CAA ACA AGC TC | |  |
| YH-DBF2-S2E-forw | GAT ACC AAC TAC GCC AAG *gag* ATT GTT GGA TCT CCA GAC TAC | |  |
| YH-DBF2-S2E-rev | GTA GTC TGG AGA TCC AAC AAT *ctc* CTT GGC GTA GTT GGT ATC | |  |
| YH-DBF2-S2A-forw | GGA TAC CAA CTA CGC CAA G*gc g*AT TGT TGG ATC TCC AGA CTA C | |  |
| YH-DBF2-S2A-rev | GTA GTC TGG AGA TCC AAC AAT *cgc* CTT GGC GTA GTT GGT ATC C | |  |
| 3xHA-tag constructs: |  | |  |
| YH-4096-SpeI-ATG | **act agt** ATG GCC GAC GAA GGA GTC G | |  |
| YH-4096-PacI-Stopp | **tta att aaC TA**A GAT CCC GCA ACG GGT CCC | |  |
| 3xmyc-tag constructs: |  | |  |
| YH-DBF2-AscI-ATG | **ggc gcg ccg** ATG TCT AGC TAC | |  |
| YH-DBF2-PacI-Stopp | GG**t taa tta a**CT ACA GCA TCG TAC C | |  |
| GFP-fusion constructs: |  | |  |
| YH-DBF2-AscI-ATG | **ggc gcg ccg** ATG TCT AGC TAC | |  |
| YH-DBF2-PacI-Stopp | GG**t taa tta a**CT ACA GCA TCG TAC C | |  |
| YH-4096-SpeI-ATG | **act agt** ATG GCC GAC GAA GGA GTC G | |  |
| YH-4096-PacI-Stopp | **tta att aa**A GAT CCC GCA ACG GGT CCC | |  |
| YH-6636-SpeI-ATG | **act agt** ATG GAG TCC CTA CTA TC | |  |
| YH-6636-PacI-Stopp | **tta att aa**G CTC AAC ACA CCC CC | |  |
| YH-1335-XbaI-ATG | **tct aga** ATG GCG CCG AAC C | |  |
| YH-1335-PacI-Stopp | **tta att aa**C GAC CAC CTC ATG TCC G | |  |
